# Alcohol Regulates Genes that Are Associated with Response to Endocrine Therapy and Attenuates the Actions of Tamoxifen in Breast Cancer Cells

**DOI:** 10.1371/journal.pone.0145061

**Published:** 2015-12-14

**Authors:** Nicholes R. Candelaria, Ryan Weldon, Selvaraj Muthusamy, Trang Nguyen-Vu, Sridevi Addanki, Paule-Helena Yoffou, Husna Karaboga, Alicia M. Blessing, Lakshmi Reddy Bollu, Rajesh C. Miranda, Chin-Yo Lin

**Affiliations:** 1 Center for Nuclear Receptors and Cell Signaling, Department of Biology and Biochemistry, University of Houston, Houston, Texas, United States of America; 2 Department of Biology and Biochemistry, University of Houston, Houston, Texas, United States of America; 3 Department of Neuroscience and Experimental Therapeutics and Women's Health in Neuroscience Program, College of Medicine, Texas A&M Health Science Center, Bryan, Texas, United States of America; Roswell Park Cancer Institute, UNITED STATES

## Abstract

Hereditary, hormonal, and behavioral factors contribute to the development of breast cancer. Alcohol consumption is a modifiable behavior that is linked to increased breast cancer risks and is associated with the development of hormone-dependent breast cancers as well as disease progression and recurrence following endocrine treatment. In this study we examined the molecular mechanisms of action of alcohol by applying molecular, genetic, and genomic approaches in characterizing its effects on estrogen receptor (ER)-positive breast cancer cells. Treatments with alcohol promoted cell proliferation, increased growth factor signaling, and up-regulated the transcription of the ER target gene *GREB1* but not the canonical target *TFF1/pS2*. Microarray analysis following alcohol treatment identified a large number of alcohol-responsive genes, including those which function in apoptotic and cell proliferation pathways. Furthermore, expression profiles of the responsive gene sets in tumors were strongly associated with clinical outcomes in patients who received endocrine therapy. Correspondingly, alcohol treatment attenuated the anti-proliferative effects of the endocrine therapeutic drug tamoxifen in ER-positive breast cancer cells. To determine the contribution and functions of responsive genes, their differential expression in tumors were assessed between outcome groups. The proto-oncogene *BRAF* was identified as a novel alcohol- and estrogen-induced gene that showed higher expression in patients with poor outcomes. Knock-down of *BRAF*, moreover, prevented the proliferation of breast cancer cells. These findings not only highlight the mechanistic basis of the effects of alcohol on breast cancer cells and increased risks for disease incidents and recurrence, but may facilitate the discovery and characterization of novel oncogenic pathways and markers in breast cancer research and therapeutics.

## Introduction

This year, more than 230,000 women in the US will develop breast cancer, currently one of the most common causes of cancer deaths in American women (Cancer Facts and Figures, American Cancer Society, 2014). A better understanding of risk factors involved in the development of breast cancer may provide more effective preventative measures as well as more targeted therapeutics. Many environmental factors are known to increase breast cancer risk, including modifiable behaviors such as alcohol consumption. Epidemiological studies have strongly linked alcohol consumption to increased breast cancer risk [1–4]. Moreover, these studies also show that breast cancer risk is positively correlated with the amount of alcohol consumed. Alcohol consumption also positively correlates with increases in breast area covered by dense parenchymal tissue and decreased β-carotene circulation, parameters which are individually known to result in increased breast cancer risk [5–8]. Furthermore, some gene product mutations (such as GSTM1) potentiate the risk for alcohol-associated cancers [9]. Given the popularity of alcohol consumption among women in the United States and a significant number of those with alcohol use disorder, alcohol consumption is a key modifiable factor in the development of breast cancer.

Alcohol-associated breast cancers tend to be estrogen receptor (ER)-positive and progesterone receptor (PR)-positive [5, 10–13]. Studies examining the potential effects of alcohol consumption on the amount of circulating estrogens in the body have failed to identify a consistent correlation, suggesting that alcohol likely mediates more direct effects on signaling mechanisms in the breast to promote carcinogenesis [14, 15]. It has been shown that alcohol stimulates proliferation, up-regulates ERα and aromatase expression, and attenuates BRCA1 expression in ER+ cell lines [16, 17]. Furthermore, it has been previously shown that alcohol up-regulates polymerase III specific genes, and that this effect is countered by treatment with ER antagonists [18, 19]. Alcohol has also been shown to increase the migration and invasion of breast cancer cell lines, which could be mediated through decreased E-cadherin expression, or up-regulated matrix metalloproteinase secretion [20, 21]. Conversely, alcohol has been shown to suppress lung metastasis of 4T1.2 breast cancer cells, which are ER- [22]. These results are difficult to interpret due to the tendency of alcohol-associated cancers to be ER+/PR+. However, another study shows that alcohol increases lung metastasis of the ER+ MADB106 breast cancer cells [23]. These experiments were performed in male rats, but suggest that alcohol may regulate breast carcinogenesis in an estrogen-dependent manner. ER and PR are markers of estrogen-dependent tumor growth and sensitivity to endocrine therapy with selective estrogen receptor modulators (SERMs) or aromatase inhibitors which block estrogen production [24]. Patients, especially postmenopausal women, who consumed alcohol while receiving endocrine therapy had a higher risk of recurrence [25]. In context of the epidemiology, It is estimated that 50% of women with breast cancer drink at least some alcohol (> 0.5 g/day), which represents a very large population and suggests a potential interaction between endocrine therapy and alcohol *in vivo* [25]. The full extent of the impact of alcohol on ER-regulated and ER-independent mechanisms remains to be determined, including interactions between alcohol, estrogen, and SERMs used to treat hormone-dependent breast cancers. In this study, we investigated the effects of alcohol on growth factor and estrogen signaling, gene regulatory networks involved in clinical outcomes in breast cancer patients, the effects of alcohol on tamoxifen response in ER+ cell lines, as well as the functions of alcohol-regulated genes in breast cancer cell proliferation.

## Materials and Methods

### Cell Culture

Three standard human breast cancer cell lines were selected for use in these studies: MCF-7, T47D, and MDA-MB-231, (American Type Culture Collection, Rockville, MD, USA). MCF-7 cells were grown in high glucose Dulbecco’s modified Eagle’s medium buffered in HEPES (Invitrogen, Carlsbad, CA, USA). The media were supplemented with 10% fetal bovine serum (Hyclone, Logan, UT, USA). T47D and MDA-MB-231 cells were grown in DMEM/F12 (Invitrogen) containing HEPES and glutamine. These cells were further supplemented with 10% FBS (Hyclone). Cells requiring estrogen-depletion were washed in PBS and grown in DMEM or DMEM/F12 lacking phenol and supplemented with 10% charcoal/dextran filtered fetal bovine serum (Hyclone).

### Cell Proliferation Assays, Cell Treatments, and Gene Knockdowns

Cells were treated with 10 nm 17β-estradiol (Sigma-Aldrich, St. Louis, MO, USA), 500 nm 4-hydroxytamoxifen (Tocris Bioscience, Bristol, UK), ethanol, or with DMSO as a vehicle. Cell proliferation was measured in one of two ways. Trypan blue exclusion assays were used to manually count cells using a hemocytometer. Otherwise, cell proliferation was measured using a standard MTS reagent, CellTiter96 Aqueous One Solution (Promega, Madison, WI, USA), according to the manufacture’s standard protocol. For combination treatment experiments, 7500 MCF-7 or T47D cells were seeded in a 96-well format, whereas 5000 MDA-MB-231 cells were similarly seeded for experimentation. Statistical analysis of these experiments was carried out using a standard two-tailed Student’s t-test. All experiments were performed in triplicate. BRAF knockdown was accomplished by transfecting breast cancer cell lines with one of two targeting siRNAs (BRAF siRNA 1: J-003460-12-0005, BRAF siRNA 2: J-003460-13-0005) following the standard manufacturer’s protocol (Thermo Scientific Dharmacon, Lafayette, CO, USA). Scrambled siRNA from the same manufacturer were utilized as negative controls. In these experiments, 5000 MCF-7 cells were seeded into a 96-well format for knockdown and subsequent MTS assays.

### Western Blotting

Cells were starved of estrogen for 72 hours prior to indicated treatment conditions for 24 hours. Cells were then lysed in standard RIPA lysis buffer. Protein concentrations were determined with Qubit Protein Assay Kit (Invitrogen). 100 μg of protein was loaded into 10% polyacrylamide gels. After separation, the proteins were then applied to PVDF transfer membranes (Thermo Fisher Scientific, Rockford, IL, USA). After transfer, the membranes were blocked in TBST with 10% dissolved nonfat milk. After blocking, the membrane was probed with antibodies directed against pERK1/2 (Cell Signaling, Danver, MA, USA), ERK1/2 (Cell Signaling), BRAF (Santa Cruz), or GAPDH dissolved in 1% milk/TBST for 4 hrs to overnight. Membranes were washed of unbound or non-specific antibody and reprobed with horseradish peroxidase (HRP) specific secondary antibodies for 1 hr. Following a second wash, the film was exposed to ECL reagent (Thermo Fisher Scientific), to allow for their detection by blue autoradiographic film. All western blot experiments were carried out in biological triplicates. Fold change quantification in protein levels was analyzed using the densitometric analysis package in ImageJ software (version 10.2) [26].

### Illumina Bead Chip Arrays and Data Analysis

Total RNA from MC7-7 cells was isolated with RNeasy columns (Qiagen). 250 ng of RNA was converted to cRNA using the Illumina TotalPrep-96 RNA Amplification kit (Ambion, Carlsbad, CA,USA). Next, cRNA from the amplification kit was hybridized to the Illumina Whole-Genome Gene Expression Direct Hybridization Microarray (Illumina, San Diego, CA, USA). The arrays were imaged in Illumina BeadArray Reader software, and were then further processed in BeadStudio software (Illumina). Signal values from unambiguous probes were local background corrected and data across arrays were quantile normalized using the lumi package from Bioconductor (https://www.bioconductor.org). Differentially expressed genes were determined by the limma package and p-values were false discovery rate corrected by the Benjamini-Hochberg procedure in R (http://r-project.org/). Genes with a correct p-value less than 0.05, as well as fold change values in excess of 1.1, were used to populate a list of responsive genes for data mining. Gene ontology and pathway analysis of responsive genes were performed using Pathway Studio (Ariadne Genomics, Rockville, MD). Fisher’s exact test was used to determine statistically enriched pathways less than the standard p-value cutoff of 0.05. Gene Set Enrichment Analysis (GSEA) was performed according to the instructions from the developer website (http://www.broadinstitute.org/gsea/doc/GSEAUserGuideFrame.html) and the responsive genes were compared to curated gene sets from the Molecular Signatures Database (MSigDB). For false discovery correction, significant gene sets were defined as those with familywise error rate (FWER)-corrected p-values of <0.05. The microarray data have been uploaded to the Gene Expression Omnibus repository and will be available to the public following publication (GSE66406).

### Quantitative PCR

RNA from treated cells was extracted using the RNeasy Kit (Qiagen). Then, 0.5 μg of RNA was reverse transcribed using SuperScript III Reverse Transcriptase System (Invitrogen). Quantitative PCR was done on a 7500 Fast Real-Time PCR system (Applied Biosystems, Carlsbad, CA, USA) using Fast SYBR Green Master Mix (Applied Biosystems). Primer BLAST was used to generate primers pairs for gene expression analysis (S1 Table). The ΔΔCt method was used to calculate fold changes between treatment conditions by normalizing to 36B4, a housekeeping gene (*36B4 forward* 5′-GTGTTCGACAATGGCAGCAT-3′; *36B4* reverse, 5′-GACACCCTCCAGGAAGCGA-3′).

### Survival Analysis

Clinical microarray gene expression data generated from a cohort of breast cancer patients in Uppsala, Sweden were used to correlate responsive alcohol genes with disease parameters and outcomes [27]. Dendrograms were generated with Eisen Cluster and Treeview software. Survival analyses were generated using the survival plot functions (log-rank test) of Mathematica software. No consent or institutional review is required for this data as the analyses were based on previously published and publically available data.

## Results

### Ethanol promotes estrogen and growth factor signaling mechanisms in breast cancer cell lines

**Fig 1:**
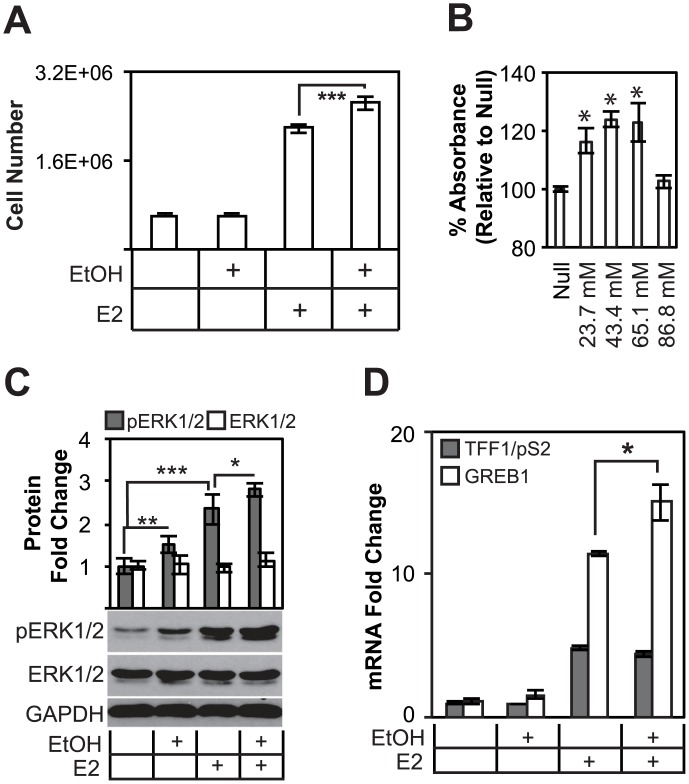
Alcohol increases cell proliferation in an estrogen-dependent manner, promotes the activation of ERK1/2, as well as known ER target genes. (A), Trypan blue exclusion assays demonstrate that estrogen potentiates cell proliferation increases by alcohol. DMSO treated cells are not statistically different from DMSO and alcohol cotreatment. (B), MTS assays measure statistically significant increases in metabolic rate at 21.7, 43.4 and 65.1 mmol/L ethanol concentrations. Treatment with 86.8 mmol/L EtOH did not result in an increase in cell proliferation. (C) Alcohol promotes the phosphorylation of ERK1/2, a key effector of growth factor signaling and of G1-S progression, regardless of estrogen treatment. Quantification comprises data of experiments in triplicate. (D) The effect of alcohol was tested on ER responsive genes TFF1/pS2 and GREB1 in MCF-7 cells. Only GREB1 responds to alcohol treatment, suggesting a possible overlap between cellular estrogen signaling and alcohol response.

To optimize the study of the effects of alcohol on breast cancer cell proliferation, several parameters were first established. MCF-7 breast cancer cells were primarily used in these studies because they are derived from the breast tumor subtype most commonly associated with alcohol consumption (ER+/PR+). They are also the most frequently used ER+ cell line and the most comprehensively studied. Cells were starved of estrogen (E2) in phenol-free medium supplemented with 10% charcoal filtered FBS for 72 hours prior to the specified treatments. Drugs and hormone compounds were dissolved in DMSO instead of ethanol to independently assess the effects of alcohol on breast cancer cell biology. After starvation and treatment, we performed trypan blue exclusion assays to evaluate whether alcohol is sufficient to drive ER+ breast cancer cell line proliferation in the absence of estrogen, which is a major target in these types of breast cancer. These results showed that 21.7 mmol/L (0.1%) alcohol increased cell proliferation in MCF-7 cells only in the presence of estrogen (Fig 1A). Cells treated with ethanol in the absence of E2 did not proliferate more than cells treated without ethanol (*p* = .77, FC = 0.97). As a positive control, E2 significantly increased proliferation in ER+ MCF-7 cells over untreated cells (*p* < 0.001). Alcohol further promoted a 21% increase in cell proliferation in E2 treated cells (*p* = 0.006), demonstrating that conditions used in these studies were sufficient to evaluate breast cancer responses to alcohol. To establish the optimal working concentration of alcohol for use in functional studies, we performed a titration of alcohol concentrations in MCF-7 cells grown in cell medium containing estrogen, then subjected them to tetrazolium salt reduction assays (MTS), which measure mitochondrial metabolic rate and act secondarily as higher throughput reflections of cell number. In this assay, cells proliferated 24% more in response to 43.4 mmol/L (0.2%) ethanol and elicited the most potent response (Fig 1B) (*p* < 0.001 for 21.7, 43.4, and 65.1 mM ethanol treatments). Cells treated to 86.8 mM (0.4%) ethanol were not statistically different from untreated cells, suggesting a suppressive effect on cell proliferation at this concentration. Furthermore, 43.4 mM (0.2%) ethanol was slightly more potent than 21.7 mM (0.1%) ethanol at increasing estrogen-dependent cell proliferation. This concentration was used for the remaining cell proliferation experiments. A blood alcohol content as low as 17.4 mM (~0.08%) begins to impair normal behaviors, and is considered a binge drinking episode [28]. Furthermore, the alcohol concentrations used in the cell proliferation experiments are higher than the amount attained in average binge episodes, but are lower than the achievable blood alcohol concentrations observed in alcohol patients [29].

Critical signals for estrogen-dependent cell proliferation are ERK1/2 phosphorylation, which is mediated though increased ER target gene transcription in response to estrogen, resulting in amplified HRG/HER2 signaling, and therefore increased growth [30]. To test whether alcohol modulates these signaling mechanisms, we carried out western blot experiments on combination estrogen and alcohol treated MCF-7 cells. These experiments showed that alcohol increased ERK1/2 phosphorylation (Fig 1C). Furthermore, pERK was increased 1.49 fold (*p* = 0.01), whereas the pERK of E2 treated cells was increased 2.3 fold (*p* = 0.001). Combination E2 and ethanol (0.2%) treatments increased pERK phosphorylation 2.8 fold relative to DMSO (*p* = 0.001). Alcohol promoted ERK phosphorylation in MCF-7 cells independent of estrogen treatment, but is still required for increased cell proliferation, suggesting estrogen-dependent and–independent mechanisms of alcohol activity in breast cancer cell lines. Despite an effect by alcohol on ERK phosphorylation levels, these experimental results demonstrated that alcohol is not sufficient to promote cell proliferation in the absence of estrogen.

Our interests in the effects of alcohol on estrogen signaling are based partially on previously published studies, which have shown that alcohol regulates estrogen receptor expression and transcriptional activity [16, 31]. To confirm these results, MCF-7 cells were treated with alcohol and/or E2 and subjected to gene expression analysis. TFF1/pS2 and GREB1 are two well-known estrogen responsive genes (Fig 1D). GREB1 expression was amplified 15.09 fold in E2 and ethanol (0.1%) treated cells over DMSO. However, GREB1 was upregulated 11.46 fold in cells treated with E2 alone. This difference was statistically significant (*p* = 0.05). Expression levels of the TFF1/pS2 mRNA transcript, however, was not statistically different between E2 and E2/EtOH treated samples, suggesting that alcohol does not amplify the expression of estrogen responsive genes in a universal fashion.

### Alcohol treatment regulates genes involved in key cellular processes that are associated with patient survival and response to endocrine therapy

**Fig 2:**
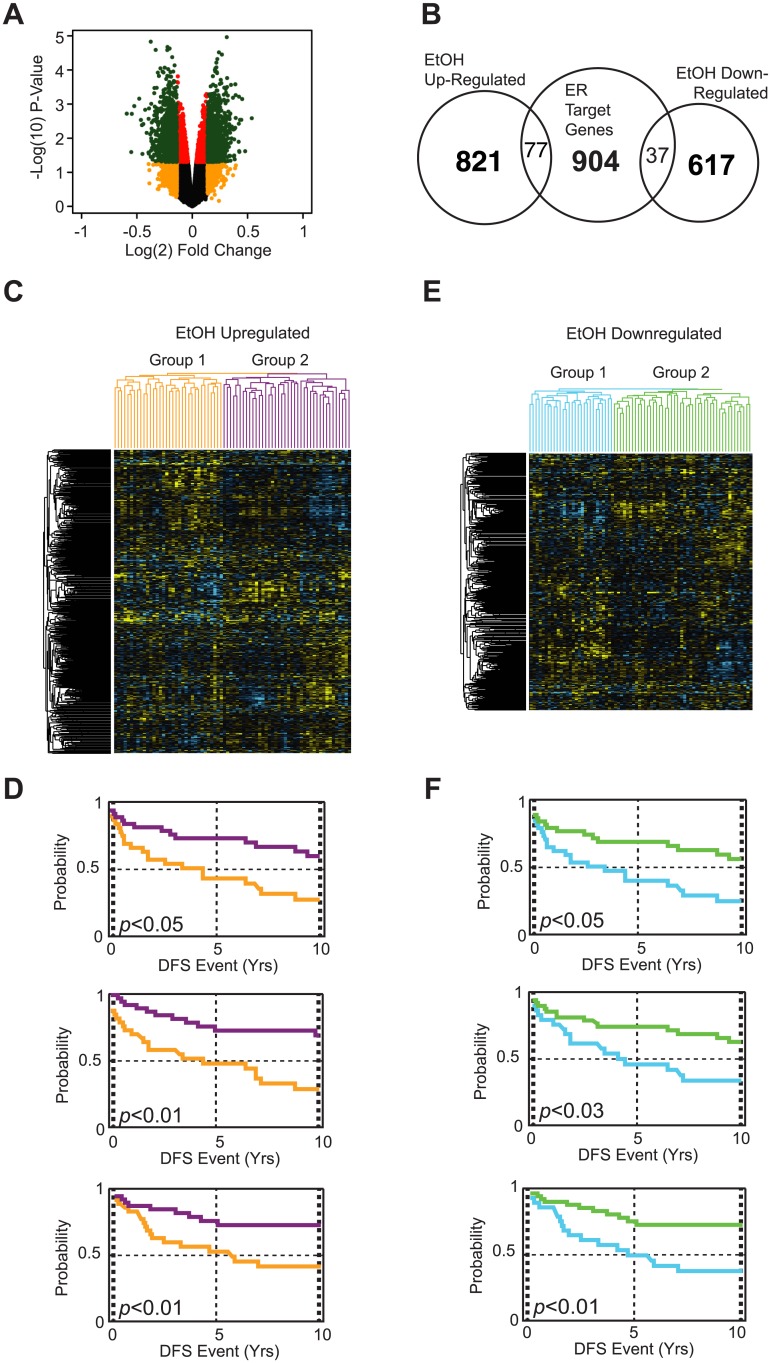
Gene networks regulated by alcohol treatment in MCF-7 cells are strongly correlated with breast cancer disease parameters. (A) A representative volcano plot that depicts all probe fold changes (log_2_) plotted against a their false-discovery corrected *p*-values (log_10_). Probes with a +/-1.1 fold change and a *p*-value < 0.05 are depicted in green and are accepted alcohol-responsive genes used in downstream analysis. (B) Representative Venn diagram demonstrating the number of up-regulated and down-regulated genes, as well as the overlap of ethanol responsive genes with ER target genes. (C) Up-regulated and (D) down-regulated alcohol responsive genes were analyzed for expression in a patient microarray (Upsalla database). Yellow colors in the expression profiles indicate up-regulated genes, whereas blue colors represent down-regulated genes. Patients were then clustered into two groups based on their gene expression profiles in (C) up-regulated and (E) down-regulated gene subsets. Parameters were correlated for DFS (disease-free survival), DMFS (mestastasis-free survival), and DSS (disease-specific survival). Survival plots of subdivided patient groups show that both (D) up-regulated and (F) down-regulated alcohol responsive genes are associated with clinical parameters and disease progression. (Patient dendrograms correspond to survival plots based on color).

To better characterize potential mechanisms of alcohol action in breast cancer cells, 0.1% ethanol-treated MCF-7 cells starved of estrogen were subjected to a genome-wide microarray analysis. Differentially expressed genes were defined by fold change cutoffs and false discovery corrected *p*-values illustrated by a volcano plot of log_10_ transformed p-values plotted against log_2_ transformed fold changes (Fig 2A). Significant fold changes (>+/-1.1) and false-discovery corrected p-values (p<0.05) accepted for further analysis are highlighted in green. The 0.1375 log2 fold-change (equivalent to 10% or 1.1-fold change in either direction) was used to further reduce the number of false-positives and increase the likelihood of validation by qPCR. It also allowed for the capture of enough data points in order to determine overlap with estrogen-responsive genes and for subsequent pathway and gene set enrichment analysis. Overall, 898 genes were upregulated, and 654 genes were down-regulated by ethanol (Fig 2B). A small portion of these genes overlapped with known ER target genes [32]. Genes that were regulated by alcohol treatment independent of a known ER binding site are termed “alcohol specific genes” in this analysis. 77 up-regulated ethanol responsive genes overlapped with the 904 previously identified ER target genes, whereas 37 down-regulated ethanol responsive genes overlapped with known ER target genes (Fig 2B). A complete list of alcohol responsive genes is included in S2 Table. A hypergeometric test revealed a statistically significant overlap between estrogen responsive genes and up-regulated alcohol regulated genes (*p*-value = 5.4x10^-8^) and nearly significant overlap between down-regulated alcohol responsive genes and estrogen responsive genes (*p*-value = 0.0964), suggesting that alcohol does not generally affect ER target genes but rather a specific subset in an apparently non-random manner. Gene ontology analysis showed that alcohol responsive genes regulated a wide variety of molecular pathways. Up-regulated alcohol-specific genes include those which governed cell cycle and apoptosis. Furthermore, the down-regulated alcohol-specific genes are also involved in apoptosis, vesicle-mediated transport, and response to oxidative stress (Table 1). The limited number of genes that overlapped with estrogen signaling (ER target genes) were involved in cellular response to p53 activity, epithelial cell maturation, and serotonin secretion (Table 2). In spite of the statistical association between alcohol and estrogen responsive genes, pathway analysis of specific gene lists failed to group into clear and cohesive consensus pathways, suggesting that the effects of alcohol on estrogen-regulated pathways are limited and gene specific (in the absence of estrogen). However, these results provide early leads into potential ER-independent mechanisms that are regulated by alcohol. To examine whether genes identified in the microarray experiments significantly overlap with other gene sets and networks, we performed gene set enrichment analysis on the alcohol responsive genes [33]. Up-regulated alcohol responsive genes are enriched for those which were also up-regulated in studies of stressed bladder cancer cells (see Table 3). Similarly, up-regulated responsive genes also showed enrichment for genes which were overexpressed in nasopharyngeal cancer as compared to normal tissues. These results suggest that exposure to alcohol may affect a number of cancer-related pathways and mechanisms.

**Table 1:** Gene ontology categories enriched in alcohol-specific responsive genes.

GO Category	Study/Category	*p*-value
***Up-regulated Genes***		
cell cycle	54/604	5.02E-11
apoptosis	62/778	9.90E-11
chromatin modification	32/262	2.02E-09
protein ubiquitination	27/220	9.98E-09
protein transport	49/602	1.32E-10
protein phosphorylation	55/743	1.84E-07
cell proliferation	38/429	1.13E-08
interspecies interaction between organisms	32/325	2.84E-08
RNA splicing	31/323	2.44E-06
protein dephosphorylation	21/166	1.33E-05
response to DNA damage stimulus	30/309	3.18E-07
***Down-regulated Genes***		
oxidation-reduction process	59/840	6.70E-11
transcription, DNA-dependent	113/2265	1.26E-08
apoptosis	51/778	3.67E-07
response to oxidative stress	19/150	4.25E-09
multicellular organismal development	66/1146	6.10E-07
vesicle-mediated transport	24/244	9.19E-08
tRNA processing	14/89	2.31E-06
viral reproduction	30/362	1.72E-07
carbohydrate metabolic process	30/369	8.14E-07
regulation of apoptosis	24/263	1.36E-06

**Table 2:** Gene ontology categories enriched in alcohol-responsive ER target genes.

GO Category	Study/Cat.	*p*-value
***Up-regulated Genes***		
positive regulation of DNA damage response, signal transduction by p53 class mediator	2 / 70	2.03E-04
regulation of bone resorption	3 / 297	2.30E-04
epithelial cell maturation	1 / 2	2.45E-04
cellular response to reactive oxygen species	1 / 2	6.87E-04
sleep	1 / 2	7.47E-04
osteoclast differentiation	1 / 2	7.47E-04
activation of adenylate cyclase activity	1 / 2	7.47E-04
cellular response to hypoxia	4 / 735	7.47E-04
epithelial cell maturation involved in salivary gland development	1 / 3	7.47E-04
serotonin secretion, neurotransmission	1 / 3	7.47E-04
***Down-regulated Genes***		
negative regulation of cell proliferation	4 / 399	2.02E-04
regulation of axonogenesis	2 / 30	2.30E-04
cellular response to starvation	2 / 31	2.45E-04
response to organic cyclic compound	3 / 234	6.87E-04
negative regulation of synaptic transmission, cholinergic	1 / 1	7.47E-04
positive regulation of calcineurin-NFAT signaling pathway	1 / 1	7.47E-04
negative regulation of hepatocyte growth factor biosynthetic process	1 / 1	7.47E-04
endocardial cushion to mesenchymal transition involved in valve formation	1 / 1	7.47E-04
mitral valve morphogenesis	1 / 1	7.47E-04

**Table 3:** Top ten gene sets enriched in the alcohol-responsive genes.

Gene Sets	FWER p-val	PubMed ID
***Up-regulated Genes***		
Genes down-regulated in fibroblasts expressing mutant forms of ERCC3 after UV irradiation	<0.001	15608684
Genes up-regulated in T1 cells (primary melanoma, sensitive to TRAIL) compared to G1 cells (metastatic melanoma, resistant to TRAIL)	<0.001	16983347
Genes up-regulated in T24 (bladder cancer) cells in response to the photodynamic therapy (PDT) stress	<0.001	17952126
Common down-regulated transcripts in fibroblasts expressing either XP/CS or TDD mutant forms of ERCC3, after UVC irradiation	<0.001	15608684
Genes up-regulated in nasopharyngeal carcinoma (NPC) positive for LMP1, a latent gene of Epstein-Barr virus (EBV)	<0.001	16912175
Genes down-regulated in NHEK cells (normal keratinocytes) by UV-B irradiation	<0.001	12771951
Genes significantly de-regulated (p < 0.05) by MIR21 in A172 cells (glioma)	0.001	18591254
Genes up-regulated in nasopharyngeal carcinoma relative to the normal tissue	0.001	16912175
Genes down-regulated in HCT116 cells (colon cancer) by expression of MIR192 or MIR215 at 24 h.	0.001	19074876
All significantly down-regulated genes in kidney glomeruli isolated from TCF21 knockout mice	0.002	16207825
***Down-regulated Genes***		
Genes co-regulated in uterus during a time course response to progesterone: SOM cluster 13	<0.001	12554760
Mitochondrial genes	<0.001	12808457
Genes whose expression was significantly and positively correlated with the number of perineuronal oligodendrocytes in the layer III of BA9 brain region	<0.001	18762803
Genes down-regulated in polysomal and total RNA samples from SW480 cells (primary colorectal carcinoma, CRC) compared to the SW620 cells (lymph node metastasis from the same individual)	<0.001	16531451
Mitochondrial genes; based on literature and sequence annotation resources and converted to Affymetrix HG-U133A probe sets	<0.001	12808457
Genes up-regulated in lymphoblastoid cells from the European population compared to those from the Asian population.	<0.001	17206142
Genes up-regulated in SKOV3ip1 cells (ovarian cancer) upon knockdown of EZH2 by RNAi	<0.001	20708159
Genes up-regulated by ESRRA only	<0.001	18974123
Genes up-regulated in HeLa cells (cervical cancer) after simultaneous knockdown of all three MBD (methyl-CpG binding domain) proteins MeCP2, MBD1 and MBD2 by RNAi	0.002	18223687
Genes up-regulated in NHEK cells (normal epidermal keratinocytes) after UVB irradiation	0.004	16434974

To determine the potential clinical significance of alcohol responsive genes in breast cancer patients, we examined their expression profiles of alcohol responsive genes in a microarray dataset of breast cancers. This dataset contains expression data from a cohort of patients with corresponding morbidity and mortality data. Only patients with ER+ breast cancers being managed by endocrine therapy were included in this analysis, due to the clinical and pathological parameters that are associated with alcohol consumption. Hierarchical clustering of these patients was performed based on the expression profiles of alcohol up-regulated and down-regulated genes. Patients were then placed into one of two patient groups based on the hierarchical clustering patterns (Fig 2C and 2E). Positive fold change values are depicted in yellow, whereas negative fold changes are shown in blue. The two groups were subsequently analyzed for disease-free survival (recurrence; DFS), distant metastasis-free survival (metastasis; DMFS), and disease specific survival outcomes (death; DSS). The up-regulated gene subset was associated with recurrence (*p* < 0.05), metastasis (*p*< 0.01), and death (*p* < 0.01) (Fig 2D). The down-regulated subset clustered patients groups with very different recurrence, metastasis, and death outcomes (*p* < 0.01, 0.025, and 0.001 respectively) (Fig 2F). Based on these survival analyses, it appears that alcohol responsive genes may serve as prognostic markers for patient response to endocrine therapy.

### Alcohol blocks tamoxifen in ER+ breast cancer cell lines

**Fig 3:**
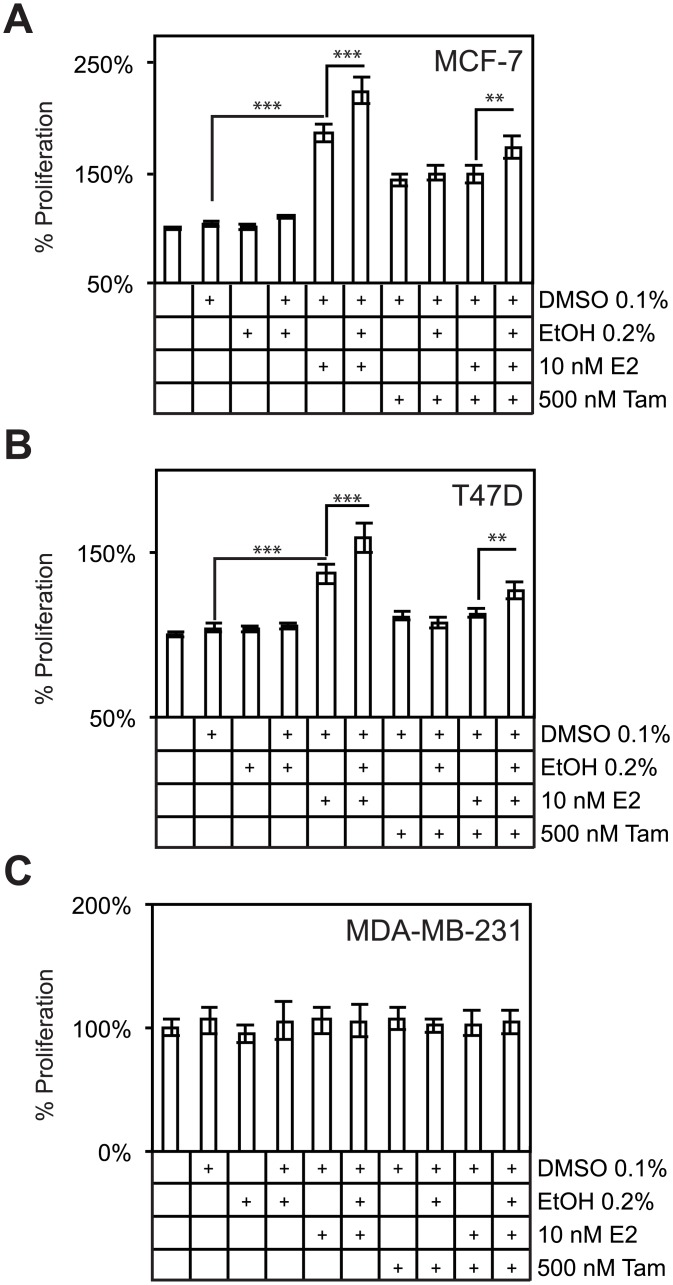
Alcohol enhances estrogen-dependent increases in cell proliferation and blocks tamoxifen attenuation of cell proliferation in MCF-7 and T47D cells. (A), MTS assays demonstrate that alcohol is able to increase measures of metabolic rate in estrogen-treated MCF7 cells. Alcohol also largely blocks a dose of tamoxifen after 72 hours of treatment, suggesting a role for alcohol in breast cancer insensitivity to SERMS. (B) Similar results were observed in another ER+ cell line, T47D. (C) MDA-MB-231 cells do not respond to estrogen, tamoxifen, or ethanol.

Due to the potential association of alcohol with response to endocrine therapy, we tested the hypothesis that alcohol may directly antagonize tamoxifen activity in breast cancer cells. In these experiments, we utilized MTS assays to measure metabolic rate in two standard ER+ cell lines (MCF-7 and T47D) and one ER- negative cell line (MDA-MB-231). As expected, the ER+ cell lines proliferated in response to E2 (80% in MCF-7 cells and 32% in T47D cells when compared to vehicle; *p* < 0.001 in both MCF-7 and T47D cells, Fig 3A and 3B). Furthermore, alcohol increased proliferation an additional 38% in MCF-7 cells and 23% in T47D cells over E2 alone (*p* ≤ 0.001 in both cell lines). As a control, tamoxifen treatment suppressed E2 induction of cell proliferation in both ER+ cell lines. To determine the association between alcohol and response to tamoxifen, ethanol co-treatment with E2 and tamoxifen increased cell proliferation 24.8% and 13.8% in MCF-7 and T47D cells respectively over E2 and tamoxifen treated cells (*p* < 0.01 in both cell lines). MDA-MB-231 cells did not respond to alcohol or ER ligands (Fig 3C). These data provide a direct link between alcohol responsive genes and previously published epidemiological data, in that expanded cell proliferation provided by estrogen and other factors is often a risk factor for the development of breast cancer [34]. These data also provide the mechanistic basis for the association between alcohol responsive genes and patient response to endocrine therapy.

### BRAF is a novel ethanol responsive gene that promotes breast cancer cell proliferation

**Fig 4:**
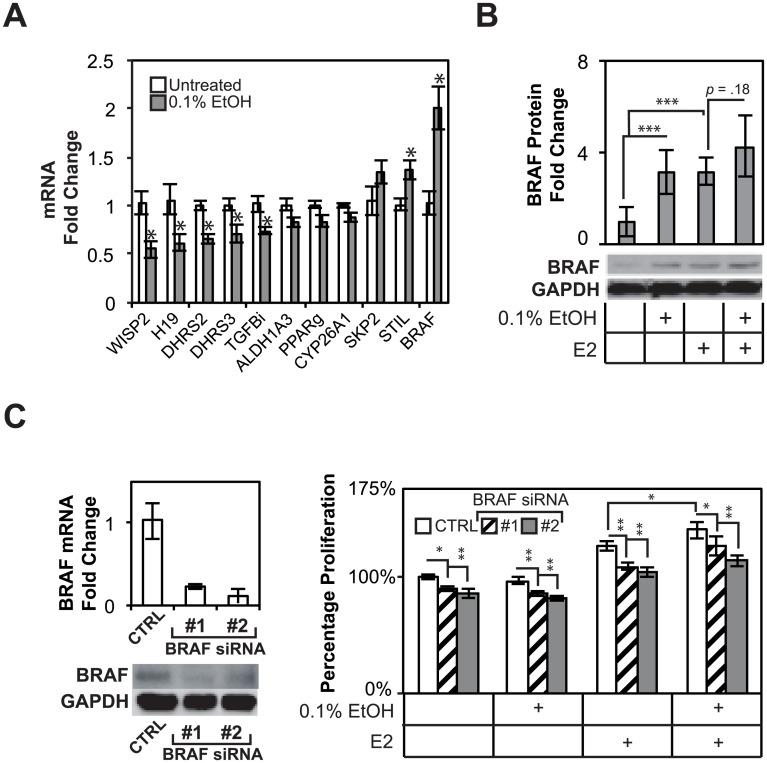
Alcohol regulates BRAF, an effector of growth factor signaling, and promotes estrogen-dependent and–independent growth. (A), Microarray validation demonstrates subtle but highly reproducible effects on gene expression of down-regulated and up-regulated genes. (B) BRAF is up-regulated at the protein level by alcohol and estrogen treatment. (C) BRAF is targetable with siRNA knockdown for functional studies. MTS assays demonstrate the anti-proliferative effect of BRAF knockdown on MCF-7 cells, suggesting that BRAF promotes basal cell proliferation in the absence of estradiol, increases estrogen-dependent growth, and potentiates some of the cell’s response to ethanol.

Previous analyses of ethanol responsive genes demonstrated a strong link between alcohol responsive genes and clinical outcomes, but involved the clustering of patients based on a large number of alcohol responsive genes (Fig 2). To ascertain the contributions of individual alcohol responsive genes to the phenotypes observed earlier, alcohol responsive genes were analyzed for their differential expression based on clinical outcomes in ER+ breast cancers treated with endocrine therapy. Single genes with statistically significant differing expression levels were identified in patients who experienced recurrence (DFS), metastasis (DMFS), or death (DSS) (Table 4). Several, alcohol responsive genes were identified in the microarray analysis and are involved in regulating cell proliferation (BRAF, SKP2, PPARG). These and other genes involved in the metabolism of alcohol were validated by qPCR (Fig 4A). The top responsive gene, BRAF, a protocol-oncogene and downstream effector of growth factor signaling and regulator of the mitogen activated protein kinase cascascadewas induced 2.00 fold over untreated cells (*p* < 0.05) at the transcript level (Fig 4A). Ethanol promoted a 3.15 fold increase in BRAF protein levels in MCF-7 cells (*p* = 0.008). Treatment with E2 increases BRAF levels 3.15 fold (*p* = 0.001), whereas E2 and EtOH treatment increased BRAF levels 4.26 fold (*p* = .004). BRAF levels for MCF-7 cells treated with E2 and alcohol were not always increased over E2 treatment alone, but a distinct trend was present (*p* = 0.18) (Fig 4B). Taken together, these data show that BRAF is a novel alcohol and estrogen responsive gene, which is overexpressed in breast cancer patients with poorer DSS parameters.

**Table 4:** Alcohol-responsive genes differentially expressed between outcome groups.

Gene	DFS	DMFS	DSS
***Up-regulated Genes***			
STIL	**0.002**	**0.004**	**0.001**
ASCL1	**0.019**	**0.035**	**0.003**
TULP4	0.244	**0.008**	**0.005**
RIF1	0.294	**0.02**	**0.005**
MIER3	0.002	**0.007**	**0.007**
BRAF	0.176	0.054	**0.014**
ID2	0.225	0.263	**0.016**
SKP2	0.505	0.081	**0.022**
TP53INP1	0.081	0.059	**0.022**
PHIP	0.594	0.65	**0.049**
***Down-regulated Genes***			
WISP2	**0.001**	**0.001**	**0.001**
DIO2	**0.013**	**0.007**	**0.002**
H19	**0.005**	**0.005**	**0.011**
PPARG	0.132	**0.048**	**0.014**
VEGFB	**0.012**	**0.023**	**0.020**
RBPMS	0.121	0.141	**0.025**
DICER1	**0.008**	**0.008**	**0.026**
DHRS2	0.676	0.633	**0.039**
ITGB5	**0.002**	**0.01**	**0.048**
VGF	**0.005**	**0.014**	**0.048**

**Fig 5:**
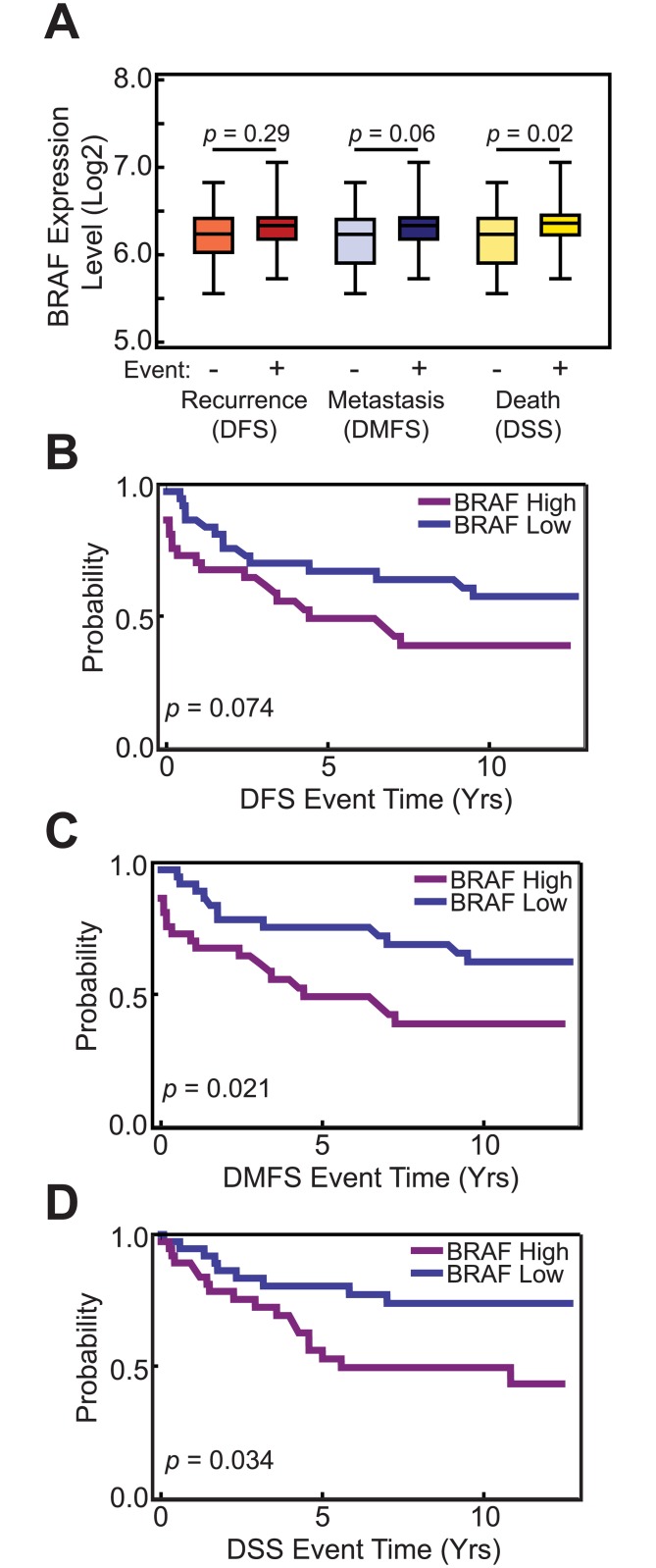
High BRAF expression levels correlated ER+ endocrine treated patients with poor prognosis and response to therapy. (A) BRAF is expressed at higher levels in patients who experience poor disease outcomes as compared to those who did not experience an adverse event. (B) BRAF expression levels separate patients into different DFS groups with nearly significant p-value (*p* = 0.074). Statistically different (C) DMFS and (D) DSS groups are observed in ER+ endocrine treated patients based on high expression levels of BRAF.

Due to its known roles in cell proliferation and oncogenesis and the activation of ERK phosphorylation in response to alcohol treatment (Fig 1), we examined the effect of BRAF on alcohol response in MCF-7 cells. First, BRAF siRNA knock-down suppressed BRAF transcript and protein levels (Fig 4C). We then performed knock-downs of BRAF and determined their effects on cell proliferation using MTS assays. Knock-down of BRAF attenuated basal proliferation rates, as well as estrogen-dependent proliferation in MCF-7 cells. Furthermore, knock-down of BRAF was able to partially attenuate alcohol (0.2%) response, especially with construct 2 (Fig 4C). Furthermore, BRAF regulated basal, as well as estrogen-dependent proliferation in MCF-7 cells. Lastly, BRAF expression is elevated in clinical samples from patients who responded poorly to endocrine therapy as determined by DFS, DMFS, and DSS (Fig 5A). Kaplan-Meier survival analysis of patients based on BRAF expression levels showed statistically significant DMFS and DSS outcomes in ER+ breast cancer patients (*p* = .02 and 0.03, respectively), where women with higher expression of the BRAF mRNA responded more poorly to endocrine therapy (Fig 5B–5D). These results identified BRAF as a novel alcohol responsive gene that is involved in breast cancer cell proliferation and whose expression is correlated with disease outcomes.

## Discussion

Approximately 60% of US women consume some alcohol annually (Women and Alcohol, National Institute on Alcohol Abuse and Alcoholism, 2015). Among those who consume alcohol, 5.7 million women have alcohol use disorders. Alcohol consumption is associated with a number of diseases, including increased risks in breast and other cancers. It is estimated that up to 5% of all breast cancers in the US and Europe are attributable to alcohol consumption [35]. Moreover, 50% of women with breast cancer consume some alcohol, and drinking is associated with an increased risk of disease recurrence in women with early stage breast cancer [25]. The aim of this study was to identify molecular pathways and mechanisms of alcohol response in ER+ breast cancer cells. We first established that alcohol increases estrogen-induced cell proliferation (Fig 1A), and these findings demonstrate that alcohol enhanced the proliferative effects of estrogen. This contrasts with other studies, which were not able to identify the link between alcohol and estrogen in cell proliferation assays [31], likely due to the lack of experiments performed on cells grown in estrogen-depleted medium containing growth factors. Furthermore, we also identified the optimal concentration of alcohol for evaluating proliferative responses in breast cancer cells while maintaining physiologically attainable levels of alcohol. The most robust proliferative response was observed in MCF-7 cells treated to 43.4 mmol/L (0.2%) alcohol (Fig 1B). To address concerns of potential cytotoxic effects of alcohol, previously published studies determined that cytotoxicity occurs at very high levels of alcohol treatment (> 425 mmol/L), concentrations which were not evaluated in our study [21]. However, we found that the alcohol-dependent proliferative concentration window (between 21.7 mmol/L and ~65.1 mmol/L alcohol) was much lower than the cytotoxic dose of alcohol (>425 mmol/L alcohol). These studies clarified the optimal parameters for studying ethanol response, which was estrogen-dependent and fell well below cytotoxic thresholds observed in other studies.

Estrogen signaling regulates and is highly integrated with growth factor signaling networks. We determined that alcohol promoted a known key regulator of estrogen-induced cell proliferation, the phosphorylation of ERK1/2, independent of estrogen (Fig 1C). Phosphorylated ERK1/2 are required for G1-S transition, and are thought to control early events in G1 by up-regulating pyrimidine synthesis, regulating protein translation, or activating transcription factors involved in subsequent cell cycle processes [36–39]. It appears from these results that alcohol promoted both estrogen- and alcohol-specific responses, as increased pERK1/2 did not result in increased proliferation in the absence of estrogen. Potential mechanisms of ERK1/2 regulation have been proposed in other studies. Increased ERK signaling could be due to the inactivation of phosphatases by reactive oxygen species (ROS) generated from alcohol detoxification, allowing for the accumulation of activating phosphorylation marks on growth factor receptors [40–42]. An alternative mechanism of the effects of alcohol on growth factor signaling pathways is that alcohol generated ROS lead to the inappropriate activation of matrix metalloproteinases, which are known to stimulate the activity of growth factor signaling ligands [20, 42]. A well-known transcriptional effector of ERK activity is activated ELK1 [43]. This factor is a potent inducer of c-*fos*, which is up-regulated in our microarray dataset (FC = 1.27). This is suggestive of increased ELK1 activity downstream of activated ERK. These results form an important link between the two critical pathways in breast cancer, growth factor signaling and estrogen signaling, which are both regulated by alcohol.

Alcohol has been shown to up-regulate the expression of an estrogen responsive luciferase reporter gene [16], an effect which was shown to require estrogen. However, the effect of alcohol on the expression of ER target genes on endogenous promoters has not been extensively explored. We showed that alcohol further increased GREB1 expression after estrogen treatment, suggesting that alcohol promotes hyper-activation of estrogen signaling in breast cancer cells (Fig 1D). TFF1/pS2, however, did not respond to alcohol treatment, possibly due to ER saturation of that promoter, negative feedback loops on transcription of the gene target, treatment time conditions, or was otherwise insensitive to the effected mechanisms of alcohol treatment. These findings contrasted with another study that described a TFF1/pS2 response to alcohol treatment, albeit the regulation was relatively subtle. We were unable to reproduce this effect in MCF-7 cells, possibly due to differences in experimental design [31].

From genome-wide microarray studies of alcohol treated cells, we first observed that a significant proportion of the genome responded to alcohol treatment (Fig 2A). Overall, the magnitude of differential expression of alcohol responsive genes was modest. This is not surprising given that alcohol is not known to be a ligand for key cell signaling pathways which can robustly activate downstream transcriptional regulatory networks. It is most likely that subtle changes across multiple pathways and gene networks are involved in the effects of alcohol on breast cancer cell biology. An example of subtle changes in gene sets having a biological impact is the study by Mootha et al., to identify genes differentially expressed in diabetic muscle samples as compared to normal controls [44]. Their inability to detect any significant changes prompted the development of GSEA [33]. The subtle changes in gene sets identified in the study were subsequently determined experimentally to be functionally important in follow-up studies [45]. Due to the depletion of estrogen in the cell culture medium, we were able to assess whether alcohol could transactivate ER target genes independent of estrogen. A hypergeometric test revealed a statistically significant overlap between up-regulated alcohol genes and known estrogen responsive genes, which suggests that a subset of estrogen responsive genes was impacted by alcohol (independently of estrogen). These data also suggest, especially in light of the estrogen-independent effect of alcohol on ERK1/2 phosphorylation, that alcohol-specific genes may potentially enhance estrogen dependent cell proliferation. To test this hypothesis, alcohol responsive genes were further analyzed for statistically enriched gene ontology categories. Cell cycle genes (CCND2, RAD17, EP300) were up-regulated in MCF-7 cells treated with alcohol (Table 1). Genes involved in protein phosphorylation (ROCK1/2, JAK2, SMAD5) and dephosphorylation (DUSP1/12, BCL2, PTP) were also regulated by alcohol treatment. As previously mentioned, cell cycle machinery is heavily dependent upon posttranslational modifications for correct regulation of growth factor signaling cascades, which could explain the enrichment of gene ontology categories involved in general protein phosphorylation. Genes involved in oxidative-reduction responses (P53, SOD1, HMOX1) and apoptotic genes (CASP2, BID, VIM) were enriched in the down-regulated alcohol specific gene subset. These data indicate that alcohol regulates a number of pathways that have known critical roles in breast carcinogenesis.

To ascertain the clinical significance of the alcohol-responsive genes, their expression profiles and association with disease outcomes were analyzed in a microarray dataset from tumors obtained from a cohort of patients who received endocrine therapy. Expression profiles of both up-regulated (Fig 2C) and down-regulated (Fig 2D) genes were strongly associated with recurrent (DFS), metastasis (DMFS), and death (DSS). It is not clear from these data what roles these genes, as a whole, may play in breast carcinogenesis, disease progression, and response to SERMs, but their association with response to endocrine therapy suggests that alcohol treatment affects the expression of a large number of genes which, at the very least, are prognostic markers of therapeutic response and may function in key molecular pathways and mechanisms. At the molecular level, normal ER activity in breast cancer cells is antagonized by SERMs, which prevents estrogen-dependent cell proliferation [46, 47]. Due to the differences in patient outcomes based on the gene expression profiles in patients of alcohol responsive genes, we suspected that alcohol might promote breast cancer cell proliferation even in the presence of tamoxifen. In agreement with this hypothesis, we determined that alcohol treatment attenuated tamoxifen suppression of cell proliferation in MCF-7 (Fig 3A) and T47D cell lines (Fig 3B). MDA-MB-231 cells did not respond to any of the treatment conditions, suggesting that ER and ER- associated factors mediate the effects of tamoxifen and alcohol. Several mechanisms of tamoxifen insensitivity have been previously identified. BRCA1 levels have been shown to be down-regulated by alcohol treatment [16]. Down-regulated BRCA1 levels lead to increased cell proliferation in the presence of tamoxifen by altering its interactions with transcriptional coregulators and alter the nature of ligand-bound ER and its downstream transcriptional responses [47]. BRCA1 was not down-regulated in our microarray analysis, which suggests that alcohol mediates its effects on BRCA1 through non-transcriptional mechanisms. Alternatively, amplified growth factor signaling can lead to increased cell proliferation in the presence of tamoxifen. In this study, we showed that growth factor signaling (pERK1/2) is activated in response to alcohol treatment, which has been shown in other studies to be up-regulated in tamoxifen resistant tumors (Fig 1C) [46]. These data together provide experimental evidence that alcohol can directly block the effects of tamoxifen and may lead to poor clinical outcomes and responses to therapy.

To further determine the mechanisms of action of alcohol in breast cancer biology, individual responsive genes were analyzed for differential expression based on clinical outcomes and response to endocrine therapy in ER+ breast cancer patients (Table 2). WISP2, for instance, is consistently down-regulated in ethanol treated cells, and has been shown to prevent migration in MCF-7 cells by up-regulating E-cadherin expression and down-regulating MMP9 activity [17]. The repressed gene dehydrogenase/reductase enzyme 2 (DHRS2) is expressed in MCF-7 cells, and is more highly expressed in luminal cells compared to basal cells, suggesting a link between higher expression of this protein and a less aggressive luminal phenotype [48]. *H19*, or maternally expressed *H19*, is a long non-coding RNA that has been shown to attenuate let-7 activity, a microRNA that regulates cell proliferation and apoptosis [49]. Deletions of the H19 mRNA have also been shown to lead to overgrowth in transgenic mouse models, possibly due to disrupted IGF-2 regulation [50]. Short-chain dehydrogenase/reductase 3 (DHRS3) has been identified as a p53 responsive gene, and functions to reduce all-trans-retinal to replenish bleached retinoids in the visual cycle [51]. DHRS3 is potently induced by retinoic acid, an antiproliferative vitamin-A derivative so alcohol may interact with vitamin-A associated pathways in breast cancer cell lines [52]. Transforming growth factor β-induced (TGFBI) is a secreted protein and is also responsive to retinoic acid treatment in MCF-7 cells and has been shown to prevent both anchorage-independent growth in MCF-7 cells and the development of metastatic lesions in mouse xenograft models [42] [53]. The SCL/TAL1 (STIL) interrupting locus gene is required for cell-cycle progression, as well as for centriole biogenesis and function [54, 55]. STIL attenuation prevents tumor growth in mouse colon cancer xenograft models [56]. The functional studies in this paper focused on BRAF, an effector of the growth factor signaling and upstream regulator of the mitogen-activated protein kinase/ERK cascade and a therapeutic target in other cancers such as melanoma [57, 58]. The observed effects of up-regulated ERK1/2 phosphorylation in response to alcohol treatment suggest a role for BRAF in alcohol responsive signaling pathways and effects (Fig 1C). BRAF is a novel alcohol- and estrogen-responsive gene, and its transcript levels were negatively correlated with patient survival and response to endocrine therapy (Fig 5). These findings suggest that alcohol inappropriately promotes sustained expression of BRAF, even in the absence of estrogen, in women who consume alcohol and may thereby mimic or enhance the effects of estrogen in increasing breast cancer risks. We determined that BRAF inhibitor sorafenib led to dramatic increases in cell proliferation (data not shown). Interestingly, this observation is in agreement with other studies that measured increased cell proliferation in the presence of BRAF inhibitors in cell lines lacking constitutive BRAF activity [59]. It is based on these observations that MCF-7 cells likely harbor wild-type BRAF. BRAF inhibitors do not appear to alter overall BRAF levels, which could explain the difference in outcomes between the use of molecular inhibitors and, in our case, siRNA knockdowns that blocked BRAF expression. A possible option for disrupting the effects of alcohol and BRAF, other than a BRAF inhibitor, is to block ERK activity using existing small molecule inhibitors. These findings not only shed light on mechanistic actions of alcohol in breast cancer but also provide insights into the cross-talk between alcohol and known and novel oncogenic pathways in breast cancer in general.

## Supporting Information

S1 Table Primers used in qPCR quantification of alcohol-responsive genes. (XLSX)

S2 Table Alcohol-responsive genes identified in microarray study. (XLSX)
